# Porous PAO/PVA Microspheres for Efficient U(VI) Capture

**DOI:** 10.3390/ma19183950

**Published:** 2026-09-17

**Authors:** Zenghui Wan, Xun Sun, Shuiyuan Zhang, Jun Yang, Long Yu, Chao Yuan, Jingshui Xu, Suhua Wang

**Affiliations:** 1 Guangdong Provincial Key Laboratory for Green Agricultural Production and Intelligent Equipment, School of Environmental Science and Engineering, Guangdong University of Petrochemical Technology, Maoming 525000, China; 2 School of Chemical Engineering, Guangdong University of Petrochemical Technology, Maoming 525000, China; 3 Guangdong Provincial Key Laboratory of Petrochemical Equipment Fault Diagnosis, School of Materials Science and Engineering, Guangdong University of Petrochemical Technology, Maoming 525000, China

**Keywords:** uranium extraction, coordination interaction, adsorption capacity, reusability, selectivity

## Abstract

The development of high-performance adsorbents with porous structure is very important for efficient uranium extraction from complex aqueous media. Here, porous poly (amidoxime)/poly (vinyl alcohol) (PAO/PVA) microspheres were fabricated by liquid nitrogen-assisted molding and subsequent vacuum freeze-drying procedures. The as-prepared composites with a PAO:PVA mass ratio of 4:1 have the superior adsorption capability at an optimal pH of 6. Its theoretical maximum adsorption capacity was estimated to be 330.2 mg g^−1^ using the Langmuir model. It also exhibited good reusability for U(VI) uptake with a high capacity retention of more than 85% after five consecutive adsorption–desorption cycles. In simulated seawater containing competing ions, the PAO/PVA microspheres can preferentially capture U(VI), achieving the highest adsorption capacity of 5.06 mg g^−1^, largest distribution coefficient of 65.71 L g^−1^ and greatest removal efficiency of 77%. The mechanism of PAO/PVA for U(VI) adsorption was further studied by Fourier transform infrared spectroscopy (FT-IR) and X-ray photoelectron spectroscopy (XPS) measurements. It was found that the N/O donor atoms derived from amidoxime were the main binding sites for U(VI). This work may provide motivation for the rational design and construction of efficient PAO-based composites for uranium adsorption.

## 1. Introduction

Nuclear energy has been attracting considerable attention due to its low-carbon characteristics, and a steady uranium supply is of significant importance for its long-term development. The oceans contain approximately 4.5 billion tons of dissolved uranium, which is more than 1000 times the identified uranium reserves on land. However, the uranium concentration in seawater is only about 3.3 μg L^−1^. It is therefore highly challenging to practically extract uranium from seawater due to the ultralow concentration, stable uranyl-carbonate speciation and dynamic marine conditions [1,2,3,4,5].

It is widely accepted that poly (amidoxime) (PAO) has become a benchmark sorbent for uranium extraction from seawater, owing to its good selectivity and high adsorption capacity, as well as its strong coordination ability with uranyl species [2,3,4,5,6,7]. However, conventional PAO-based materials still suffer from slow mass transfer, difficult recovery and the aggregation of molecular chains when directly used as powders or weak gels, leading to poor performance for uranium capture in complex marine environments [8,9]. Constructing PAO/poly (vinyl alcohol) (PAO/PVA) composite materials is believed to be an effective strategy to improve uranium extraction efficiency because their good hydrophilicity and abundant hydroxyl groups of PVA can stabilize polymer networks and reorganize hydrogen-bonding interactions. For example, a macro-channel PAO-PVA hydrogel membrane has been successfully synthesized by the double cross-linking strategy and displayed exceptional performance for uranium adsorption [10]. The PAN-AO/PVA composites, prepared through electrospinning and subsequent amidoximation treatment, also exhibited high adsorption capacity for uranium extraction from seawater [11]. Despite the above progress, the utilization ratio and reusability of PAO-based adsorbents still need to be promoted urgently.

Herein, physically cross-linked PAO/PVA microspheres with hierarchical porous structure have been successfully constructed by liquid nitrogen-assisted molding and subsequent vacuum freeze-drying procedures. Benefiting from the unique spherical architecture and stable crosslinking network, the PAO/PVA microspheres exhibited excellent performance for U(VI) capture, including large adsorption capacity, high selectivity and good reusability. The effects of pH value, material ratio, contact time, initial concentration, reaction temperature and competing ions on U(VI) adsorption property were systematically studied. The chemical interaction between PAO/PVA and uranyl species was further investigated by spectroscopic techniques.

## 2. Experimental Section

### 2.1. Reagents

Poly (vinyl alcohol) (PVA, 99% hydrolyzed, 2400 polymerization), polyacrylonitrile (PAN, 99.5%, Mw ≈ 85,000), anhydrous sodium carbonate (Na_2_CO_3_, 99.99%), hydrochloric acid (HCl, 37 wt%), and uranyl nitrate hexahydrate (UO_2_(NO_3_)_2_·6H_2_O) were purchased from Shanghai Aladdin Biochemical Technology Co., Ltd. (Shanghai, China). Dimethylformamide (DMF, 99%), hydroxylamine hydrochloride (NH_2_OH·HCl, 99%), and Arsenazo III (95.0%) were obtained from Shanghai Macklin Biochemical Co., Ltd. (Shanghai, China). Sea salt was supplied by Guangzhou Deli Chemical Co., Ltd. (Guangzhou, China). All reagents were directly used without further purification.

### 2.2. Materials Characterization

The microstructure and elemental distribution of the PAO/PVA microspheres were examined by field-emission scanning electron microscopy (SEM; Hitachi Regulus 8220, Tokyo, Japan). Fourier-transform infrared (FT-IR) spectra were acquired with a Nicolet iS50 spectrometer (Waltham, MA, USA). Nitrogen adsorption–desorption isotherms were obtained at 77 K with a BSD-660 M instrument (Beijing, China). The pore size distribution was determined by the BJH (Barrett–Joyner–Halenda) method. X-ray photoelectron spectroscopy (XPS) measurements were performed on a Thermo Scientific K-Alpha instrument (Waltham, MA, USA). The concentrations of metal ions were determined by inductively coupled plasma mass spectrometry (ICP-MS; Agilent 7700, Santa Clara, CA, USA).

### 2.3. Sample Preparation

#### 2.3.1. Preparation of PAO

PAO was synthesized by the amidoximation of PAN. A total of 7.64 g NH_2_OH·HCl was first dispersed in 100 mL DMF, followed by the addition of 6.0 g Na_2_CO_3_ and 1.52 g NaOH. The mixture was vigorously stirred at 45 °C for 2 h. Then, 4 g PAN powder was added and stirred at 65 °C for 24 h. Additionally, 2.65 g NH_2_OH·HCl was dissolved in 30 mL DMF, followed by the addition of 2.4 g Na_2_ CO_3_ and 0.541 g NaOH. After stirring for 10 min, this solution was added to the PAN-containing mixture, and amidoximation was continued at 65 °C for another 24 h. The as-obtained precipitate was collected by centrifugation, repeatedly washed with deionized water until pH ≈ 7, and then dried under vacuum at 55 °C to obtain PAO powder.

#### 2.3.2. Preparation of PAO/PVA Microspheres

A total of 10 g PVA was first dissolved in 100 mL deionized water under magnetic stirring at 95 °C for 12 h. Then, 2 g PAO was dissolved in 20 mL 0.3 mol L^−1^ NaOH solution, followed by the addition of 5 mL as-prepared PVA solution to obtain a homogeneous precursor. The above precursor was dropped into liquid nitrogen by using a syringe at a rate of 500 μL min^−1^ to form spherical samples, which were finally transferred into a vacuum freeze-dryer and dried for 48 h to obtain the PAO/PVA microspheres.

### 2.4. U(VI) Adsorption Experiments

The uranium stock solution (1.0 g L^−1^) was prepared by dissolving 2.1091 g UO_2_(NO_3_)_2_·6H_2_O in 10 mL 0.1 mol L^−1^ HNO_3_ and a certain amount of deionized water. The solution was diluted to 50 mg L^−1^ for further use, and the pH was adjusted to 3~9 with 0.1 mol L^−1^ HNO_3_ or NaOH solution. A known mass of PAO/PVA was added to the U(VI)-containing solution in a conical flask, followed by agitation in a reciprocating water bath oscillator for a specified contact time. After adsorption, the solid and liquid phases were separated through the 0.22 μm membrane. U(VI) in the supernatant was quantified using a UV-Vis spectrophotometer (Waltham, MA, USA) with Arsenazo III as the chromogenic reagent at a wavelength of 652 nm. The equilibrium adsorption capacity (*q_e_*, mg g^−1^) and removal efficiency (*R*, %) were calculated using Equations (1) and (2), respectively.

(1) $$q_{e}=\frac{\left(C_{0}-C_{e}\right)\times V}{m}$$

(2) $$R=\frac{\left(C_{0}-C_{e}\right)}{C_{0}}\times 100$$

where *C*_0_ and *C_e_* are the initial and equilibrium U(VI) concentrations (mg L^−1^), respectively, *V* is the solution volume (L), and *m* is the mass of adsorbent (g).

## 3. Results and Discussion

### 3.1. Structural Analysis

**Figure 1:**
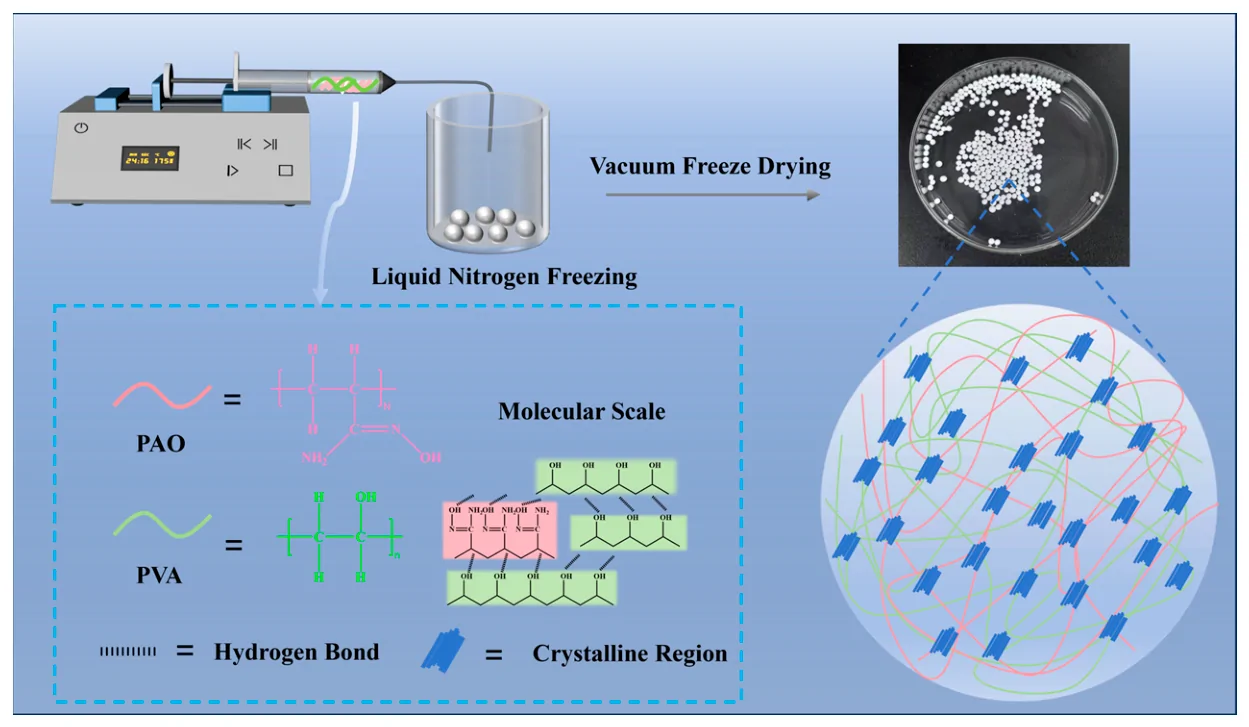
Schematic illustration of the preparation of PAO/PVA microspheres.

**Figure 2:**
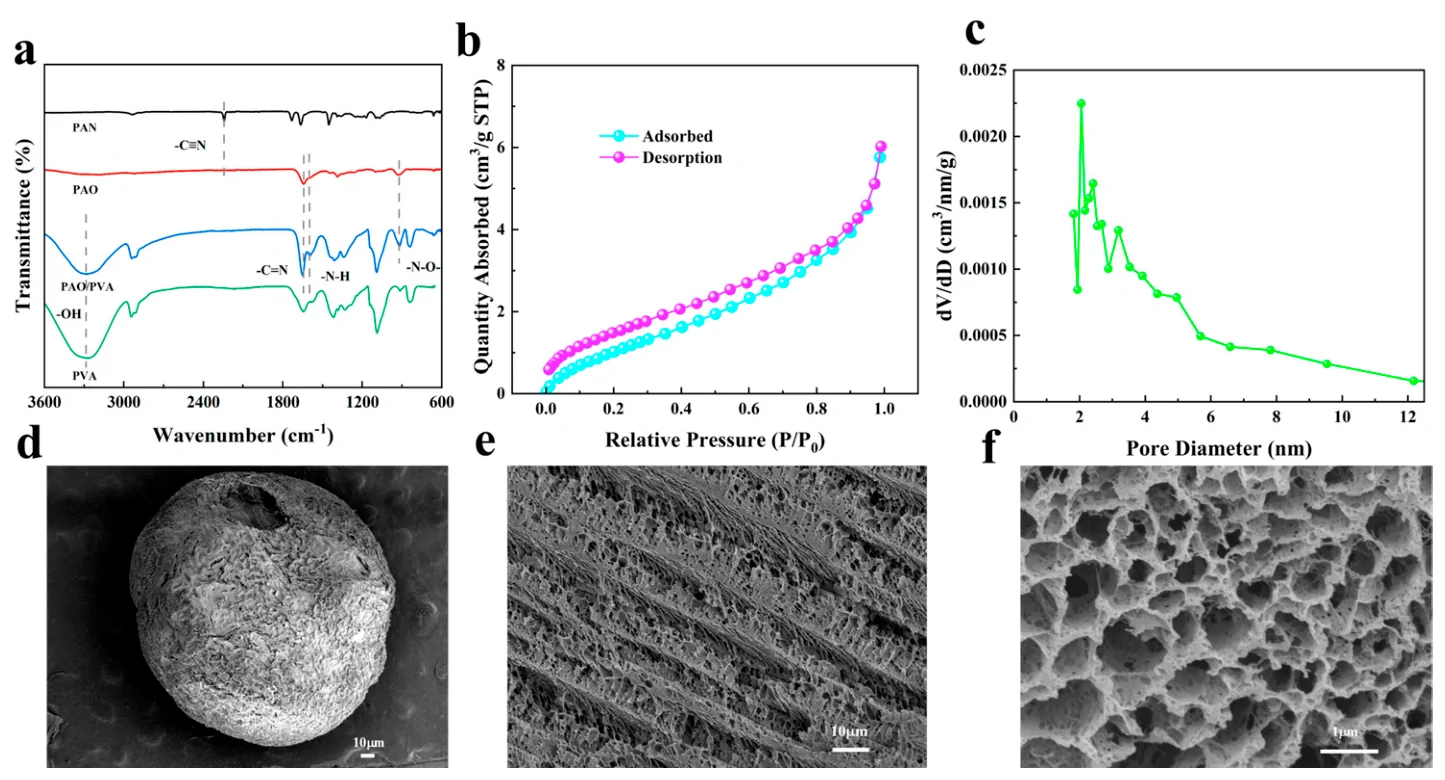
(**a**) FT-IR spectra associated with PAO/PVA formation. (**b**) N_2_ adsorption–desorption isotherm of PAO/PVA. (**c**) The corresponding pore size distribution plot. (**d**–**f**) SEM images of PAO/PVA.

The porous PAO/PVA microspheres with physical crosslinks were synthesized by liquid nitrogen-assisted molding and subsequent vacuum freeze-drying procedures (Figure 1). The successful preparation of PAO/PVA composites was first confirmed by FT-IR measurements. As shown in Figure 2a, the characteristic C≡N band of PAN at 2243 cm^−1^ vanished after amidoximation, while three new bands appeared at 1658 cm^−1^ (C=N), 1388 cm^−1^ (C-N) and 938 cm^−1^ (N-O), respectively, indicating the conversion of PAN to PAO [8,12,13,14,15]. Moreover, a broad band associated with O-H stretching was observed at around 3285 cm^−1^, which suggested the successful incorporation of PVA and PAO. The ^13^C NMR spectra were also indicative of the formation of PAO (Figure S1). The signal of -C≡N at 120.54 ppm disappeared after the reaction, whereas a new signal of -C(=N-OH)-NH_2_ at 162.91 ppm could be assigned to the amidoxime [6,16,17,18]. The above spectral features demonstrated the successful amidoximation of PAN, which was crucial for uranium capture, because the N/O donor atoms of the amidoxime groups can coordinate with uranyl species.

The N_2_ adsorption–desorption isotherms of PAO/PVA microspheres exhibited a type-IV profile with an H_3_-type hysteresis loop (Figure 2b), indicating the existence of the mesoporous configuration. Its specific surface area derived from the isotherms was further calculated to be 7.31 m^2^ g^–1^, with a pore size of 2–12 nm (Figure 2c). It is generally believed that the porous structure can contribute well to the adsorbate diffusion and mass transfer and thus to an improvement in performance [8,9,10,19,20]. SEM was then used to investigate the morphology and microstructure of the as-prepared samples. Figure 2d displays intact spherical particles with diameters of approximately 100–200 μm. The surface of PAO/PVA microspheres is rough and porous, while the interior exhibits an interconnected honeycomb-like framework with open channels of approximately 0.5–2 μm (Figure 2e,f). Such micron-scale channels are favorable to facilitate solution penetration and shorten diffusion distance [8,9,10,15,19]. As shown in Figure S2, TGA-DTG analysis indicated that the PAO/PVA composites can retain their framework well below the main thermal decomposition temperature of 262.02 °C. This is much higher than the typical temperature (25–60 °C) for adsorption experiments, demonstrating the adequate thermal stability for U(VI) capture. The mass loss at lower temperature might be associated with physically adsorbed water because the PAO/PVA composites contained abundant hydrophilic groups, such as O-H and N-H.

### 3.2. Adsorption Experiments

#### 3.2.1. Effect of Solution pH

**Figure 3:**
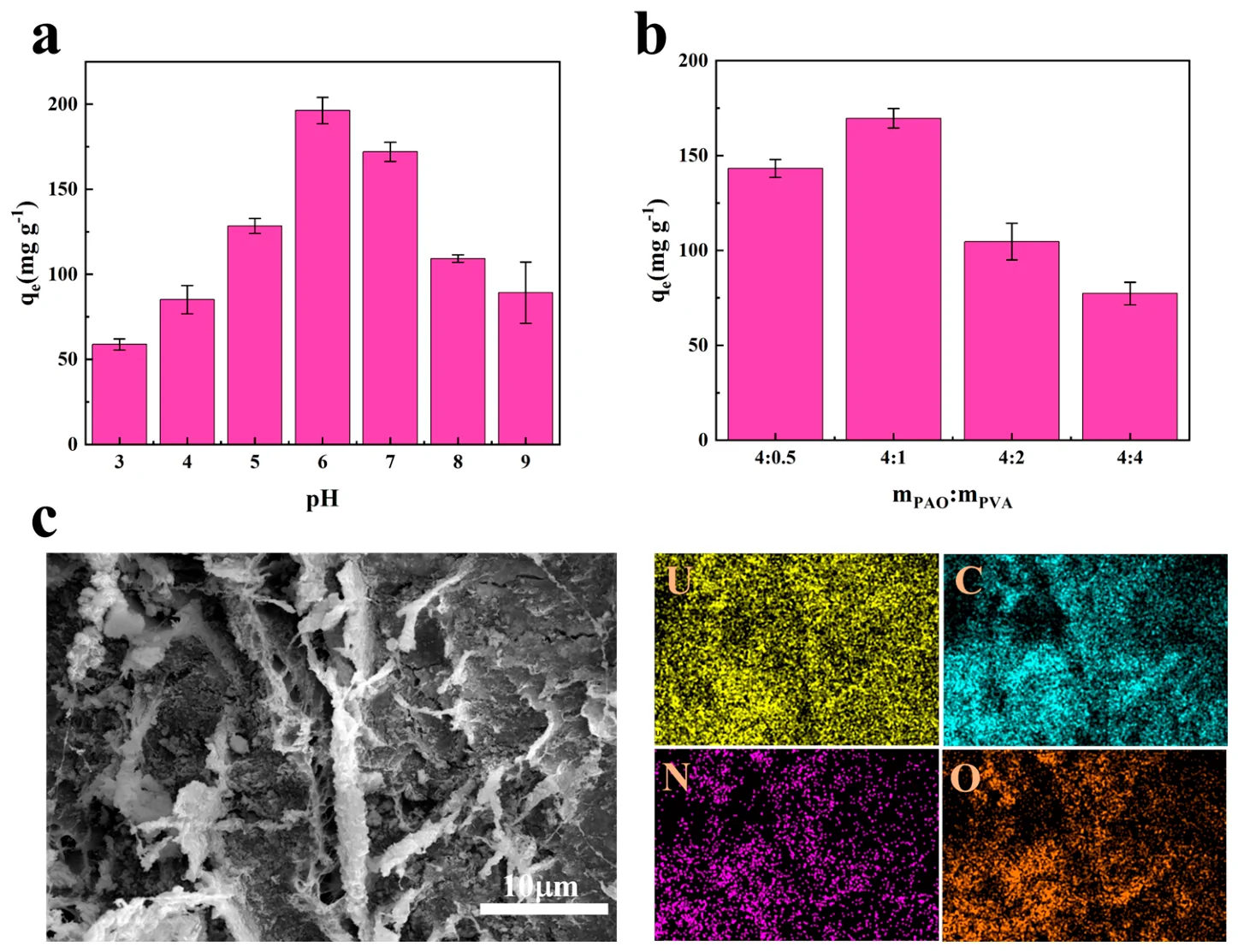
(**a**) Effect of pH on U(VI) adsorption (*C*_0_ = 50 mg L^−1^; *V* = 40 mL; *m* = 5 mg; *t* = 24 h; *T* = 298 K). (**b**) Effect of the PAO:PVA mass ratio on U(VI) adsorption under the same conditions. (**c**) SEM and elemental mapping images of PAO/PVA after U(VI) adsorption.

It is widely accepted that pH is a key parameter for governing U(VI) adsorption performance. As depicted in Figure 3a, the adsorption capacity of PAO/PVA increased markedly as the pH increased from 3 to 6 and then decreased when the pH was further increased to 9. At low pH, the protonation of N/O donor groups results in the electrostatic repulsion between coordination sites and positively charged uranyl species. With the elevation of pH, deprotonation increases the availability of adsorption sites and thus promotes U(VI) uptake [5,6,21]. Beyond the optimum, the changes in uranyl speciation and surface state of the adsorbent can weaken the complexation. The highest adsorption capacity was observed at pH = 6, and this value was therefore selected for subsequent experiments.

#### 3.2.2. Effect of PAO/PVA Mass Ratio

The composition of PAO/PVA was further optimized to achieve favorable adsorption performance. It can be seen that the adsorption capacity increases with the introduction of PVA and reaches the maximum at a PAO:PVA mass ratio of 4:1 (Figure 3b). On the basis of adsorption optimization, this proportion was selected for subsequent experiments. A moderate amount of PVA content is expected to promote the formation of a physically cross-linked network and improve accessibility of adsorption sites [10,11]. Further increasing the PVA content reduces the fraction of amidoxime-rich PAO and lowers the density of uranium-binding sites, thus leading to poor adsorption capacity. As shown in Figure S3, the obvious color change was indicative of the successful capture of uranium. The mapping images of PAO/PVA after U(VI) adsorption further demonstrate the uniform distribution of U, C, N and O elements (Figure 3c).

#### 3.2.3. Effect of Contact Time and Adsorption Kinetics

**Figure 4:**
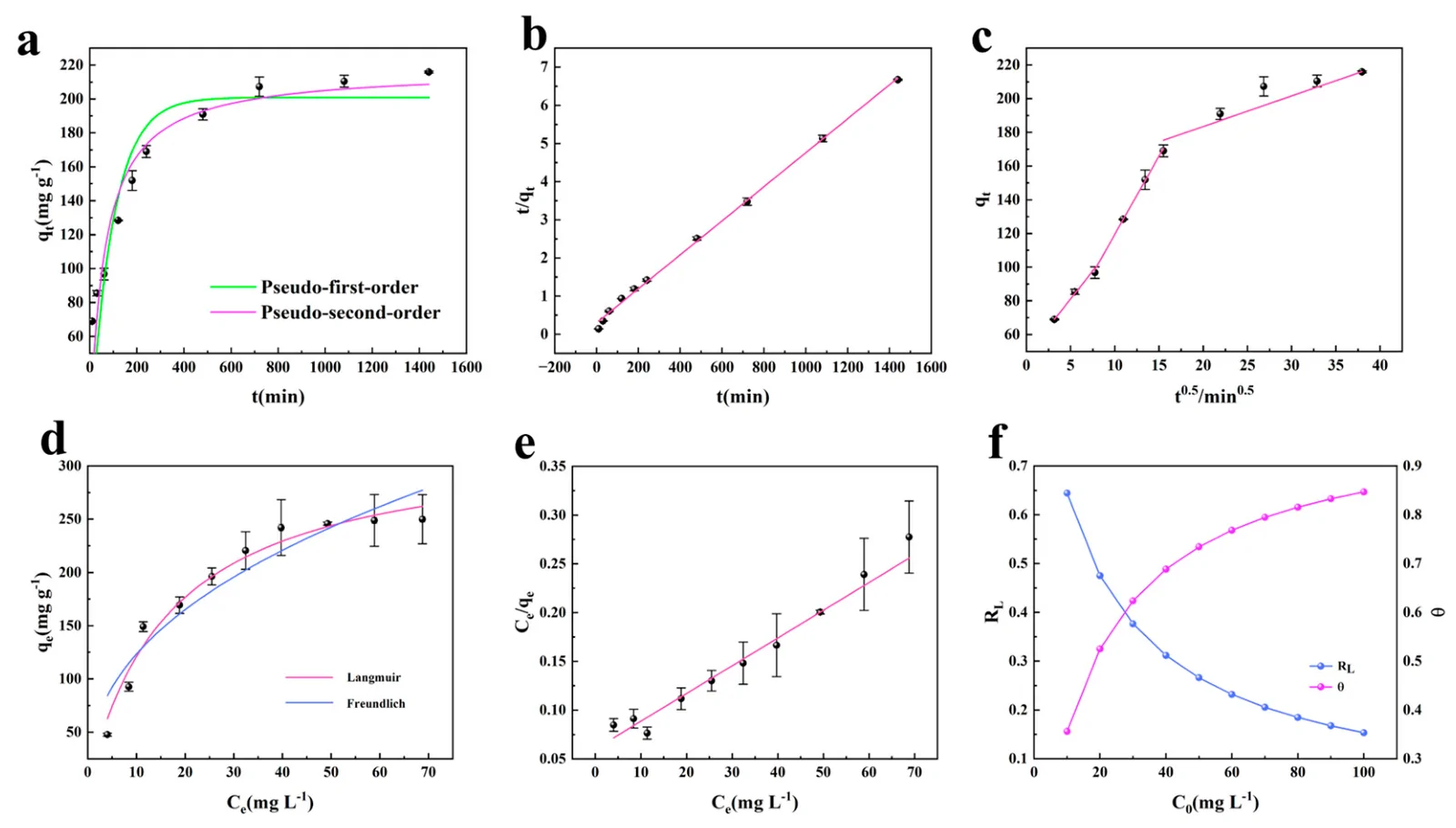
(**a**) U(VI) adsorption kinetics of PAO/PVA. (**b**) The pseudo-second-order model. (**c**) The intraparticle diffusion model (*C*_0_ = 50 mg L^−1^, *V* = 40 mL, *m* = 5 mg, *t* = 0–24 h, *T* = 298 K). (**d**) Adsorption isotherms of PAO/PVA. (**e**) The corresponding Langmuir model. (**f**) Langmuir separation factor (*R_L_*) and surface coverage (θ).

As shown in Figure 4a, the adsorption process can be divided into three stages: a rapid uptake stage (0–120 min), a slower stage (120–300 min), and an equilibrium stage after 300 min. The rapid adsorption at the initial stage can be attributed to fast transport of U(VI) to readily accessible external sites. When these sites were occupied, diffusion into the internal pores became increasingly important and the adsorption rate gradually decreased [8,10,22]. The adsorption capacity was almost constant after 300 min, which indicated that the system achieved adsorption equilibrium under the operation condition. The adsorption kinetics were evaluated by using the pseudo-first-order (PFO), pseudo-second-order (PSO), and intraparticle diffusion models (Equations (3)–(5)).

(3) $$\ln(q_{e}-q_{t})=\ln q_{e}-k_{1}t$$

(4) $$\frac{t}{q_{t}}=\frac{1}{k_{2}q_{e}^{2}}+\frac{t}{q_{e}}$$

(5) $$q_{t}=k_{i}t^{0.5}+C$$

where *t* is the contact time (min), *q_t_* and *q_e_* are the adsorption capacity at a given time and at equilibrium (mg g^−1^), respectively, *k*_1_ is the PFO rate constant (min^−1^), *k*_2_ is the PSO rate constant (g mg^−1^ min^−1^), *k_i_* is the intraparticle diffusion rate constant (mg g^−1^ min^−0.5^), and *C* is the intercept associated with the boundary layer contribution (mg g^−1^).

Among the above empirical models, the PSO model gives the highest correlation, and the calculated equilibrium capacity is very close to the experimental value (Figure 4b and Table S1). The intraparticle diffusion plot in Figure 4c exhibited three linear regions, corresponding to the multiple mass transfer steps. The first region was associated with rapid U(VI) adsorption on readily accessible sites, the second region corresponded to slower diffusion into internal pores, and the final region reflected adsorption–desorption equilibrium. It was found that the fitted lines did not pass through the origin, indicating that intraparticle diffusion was not the only rate-determining factor, and boundary-layer mass transfer could also contribute to the overall kinetics.

#### 3.2.4. Effect of Initial U(VI) Concentration and Adsorption Isotherms

As shown in Figure 4d, the equilibrium adsorption capacity increased markedly with increasing U(VI) concentration and then gradually approached a plateau. At low concentrations, abundant active sites were available for binding U(VI) ions. An increase in U(VI) concentration can strengthen the driving force for mass transfer, thus improving the site occupancy and capture performance. At higher concentrations, progressive saturation of binding sites limited further increase in adsorption capacity. The adsorption characteristics of the PAO/PVA microspheres were then investigated by using the Langmuir and Freundlich isotherm models [23] (Equations (6) and (7)) in the U(VI) concentration range of 10 to 100 mg L^−1^.

(6) $$q_{e}=q_{m}\frac{K_{L}C_{e}}{1+K_{L}C_{e}}$$

(7) qe=KFCe1n

where *C_e_* is the equilibrium U(VI) concentration (mg L^−1^), *q_e_* is the equilibrium adsorption capacity (mg g^−1^), *q_m_* is the theoretical adsorption capacity (mg g^−1^), *K_L_* is the Langmuir affinity constant (L mg^−1^), *K_F_* is the Freundlich constant [(mg g^−1^) (L mg^−1^)^1/n^], and 1/*n* is an empirical parameter associated with the favorability of adsorption.

The fitting curve is shown in Figure 4d, and the fitting results are summarized in Table S2. The Langmuir model exhibited a higher coefficient of determination (R^2^ = 0.972) than the Freundlich model (R^2^ = 0.908), indicating that it was more suitable to describe the present equilibrium data. The corresponding theoretical adsorption capacity was calculated to be 330.2 mg g^−1^. It is worth noting that the PAO/PVA in this current work exhibited competitive adsorption capacity, as compared with other advanced AO-based adsorbents for U(VI) capture (Table S3). The favorability of the Langmuir model was further evaluated using the separation factor (*R_L_*) and surface coverage (*θ*), as defined by Equations (8) and (9), respectively.

(8) $$R_{L}=\frac{1}{1+K_{L}C_{0}}$$

(9) $$\theta =\frac{K_{L}C_{e}}{1+K_{L}C_{e}}$$

where *K_L_* is the Langmuir affinity constant and *C*_0_ is the initial adsorbate concentration.

The conventional interpretation is as follows: *R_L_* > 1, *R_L_* = 1, 0 < *R_L_* < 1 and *R_L_* = 0 indicate the unfavorable, linear, favorable and irreversible adsorption, respectively. It can be seen that the current *R_L_* values were in the range of 0~1 (Figure 4f), indicating the favorable U(VI) uptake. Additionally, the *θ* values increased rapidly with increasing initial U(VI) concentration and then approached a plateau, in accord with the progressive occupation of accessible sites.

#### 3.2.5. Effect of Reaction Temperature

**Figure 5:**
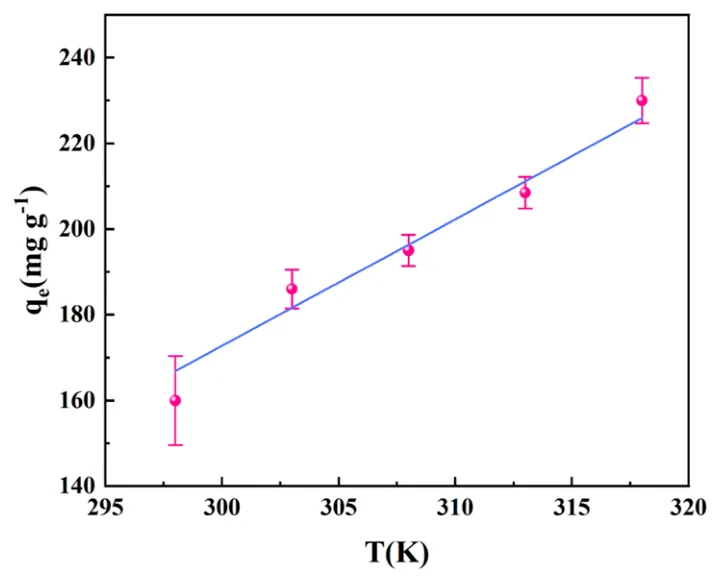
Effect of temperature on equilibrium adsorption capacity of PAO/PVA for U(VI).

The effect of temperature on U(VI) adsorption was then studied at 298, 303, 308, 313, and 318 K. As shown in Figure 5, the equilibrium adsorption capacity increased from 159.9 to 229.9 mg g^−1^ as the temperature rose from 298 to 318 K, which suggested that a higher temperature favors U(VI) uptake under the test conditions.

#### 3.2.6. Regeneration, Reusability and Selectivity of PAO/PVA

**Figure 6:**
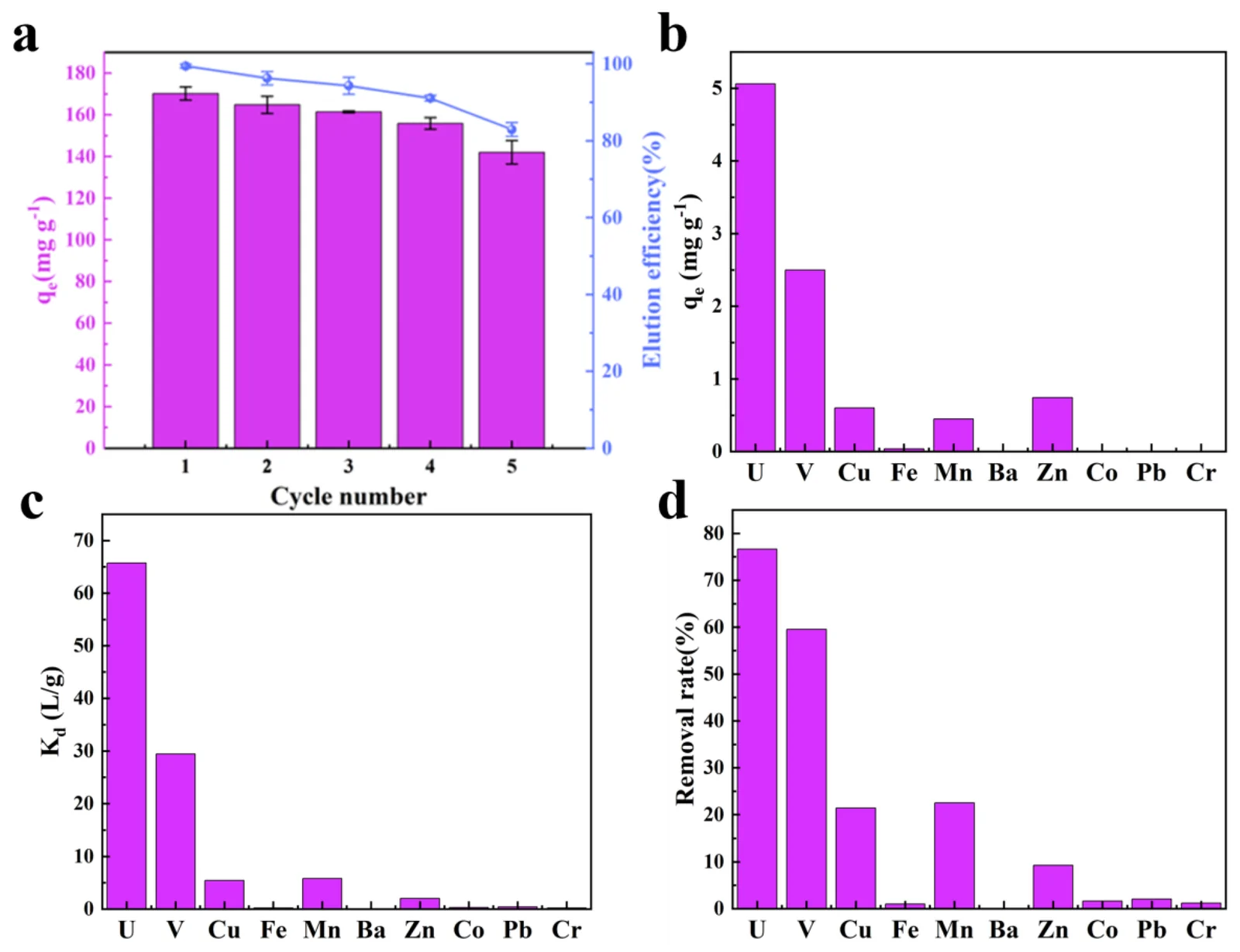
(**a**) Reusability of PAO/PVA for U(VI) capture (pH = 6, *t* = 300 min, m/V = 0.125 g L^−1^, *T* = 313 K, *C*_0_ = 50 mg L^−1^). (**b**) Adsorption capacities, (**c**) distribution coefficients and (**d**) removal efficiencies for U(VI) and competing ions.

The regeneration and reusability of PAO/PVA microspheres were evaluated over five consecutive adsorption–desorption cycles. After U(VI) adsorption, the uranium-loaded microspheres were treated with 0.5 mol L^−1^ Na_2_CO_3_, achieving a desorption efficiency above 90% (Figure S4). The regenerated adsorbent was subsequently washed to neutrality with deionized water and reused for the next adsorption. As shown in Figure 6a, more than 85% of the initial adsorption capacity was retained after five cycles, indicating the good reusability and cycling stability of PAO/PVA. In addition, the as-prepared samples could be redissolved in hot water at 95 °C to form a homogeneous polymer solution (Figure S5), which suggested the thermally reversible nature of the physically cross-linked PAO/PVA microspheres. The selectivity of PAO/PVA for U(VI) capture was further evaluated in simulated seawater containing representative competing ions (V^5+^, Ba^2+^, Cu^2+^, Fe^3+^, Co^2+^, Zn^2+^, Mn^2+^, Pb^2+^ and Cr^3+^). Their initial concentrations are listed in Table S4. Although the coexisting ions have much higher concentrations than uranium, the PAO/PVA microspheres can achieve a high U(VI) adsorption capacity of 5.06 mg g^−1^ within 24 h (Figure 6b). As can be seen, V^5+^ was the strongest competitor with an adsorption capacity of 2.5 mg g^−1^. The distribution coefficient for uranium reached up to 65.71 L g^−1^ (Figure 6c), which was much higher than those of the other ions. As displayed in Figure 6d, the U(VI) removal efficiency was also as high as 77%. These results demonstrate that the PAO/PVA microspheres can preferentially adsorb U(VI) under multicomponent conditions.

### 3.3. Adsorption Mechanism

**Figure 7:**
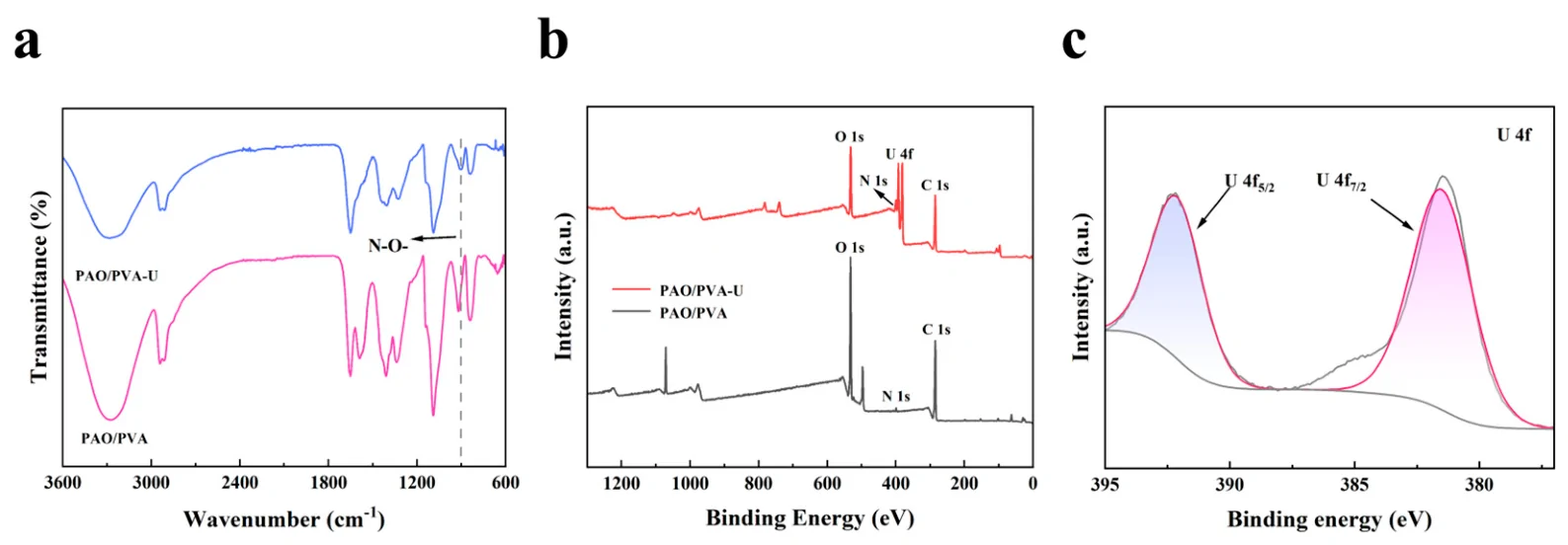
(**a**) FT-IR and (**b**) survey XPS spectra of PAO/PVA before and after U(VI) adsorption. (**c**) High-resolution U 4f spectrum of PAO/PVA after U(VI) adsorption.

The interaction between PAO/PVA and U(VI) was further investigated by SEM-EDS, FT-IR, and XPS measurements. As shown in Figure S6, the atomic percentage of uranium was determined to be 14.68% after adsorption, and a new signal associated with uranium was observed at 3.20 keV. As discussed above, the uniform distribution of U, C, N and O (Figure 3c) also confirmed the successful uptake and retention of uranium. After U(VI) adsorption, changes were observed in the region associated with N-O/uranyl vibrations at 921 cm^−1^ (Figure 7a). Compared with the uranyl vibration of UO_2_(NO_3_)_2_·6H_2_O at 960 cm^−1^, this shift might be attributed to the alteration of uranyl bonding environment [6,16,17,18]. These spectral changes indicated the strong chemical interaction between U(VI) and PAO/PVA. As shown in Figure 7b, a clear signal of U 4f was detected after adsorption. The high-resolution U 4f spectrum in Figure 7c can be deconvoluted into two peaks at 392.28 and 381.58 eV, which were assigned to U 4f_5/2_ and U 4f_7/2_, respectively. The above results further confirmed the retention of uranyl species on PAO/PVA after adsorption.

**Figure 8:**
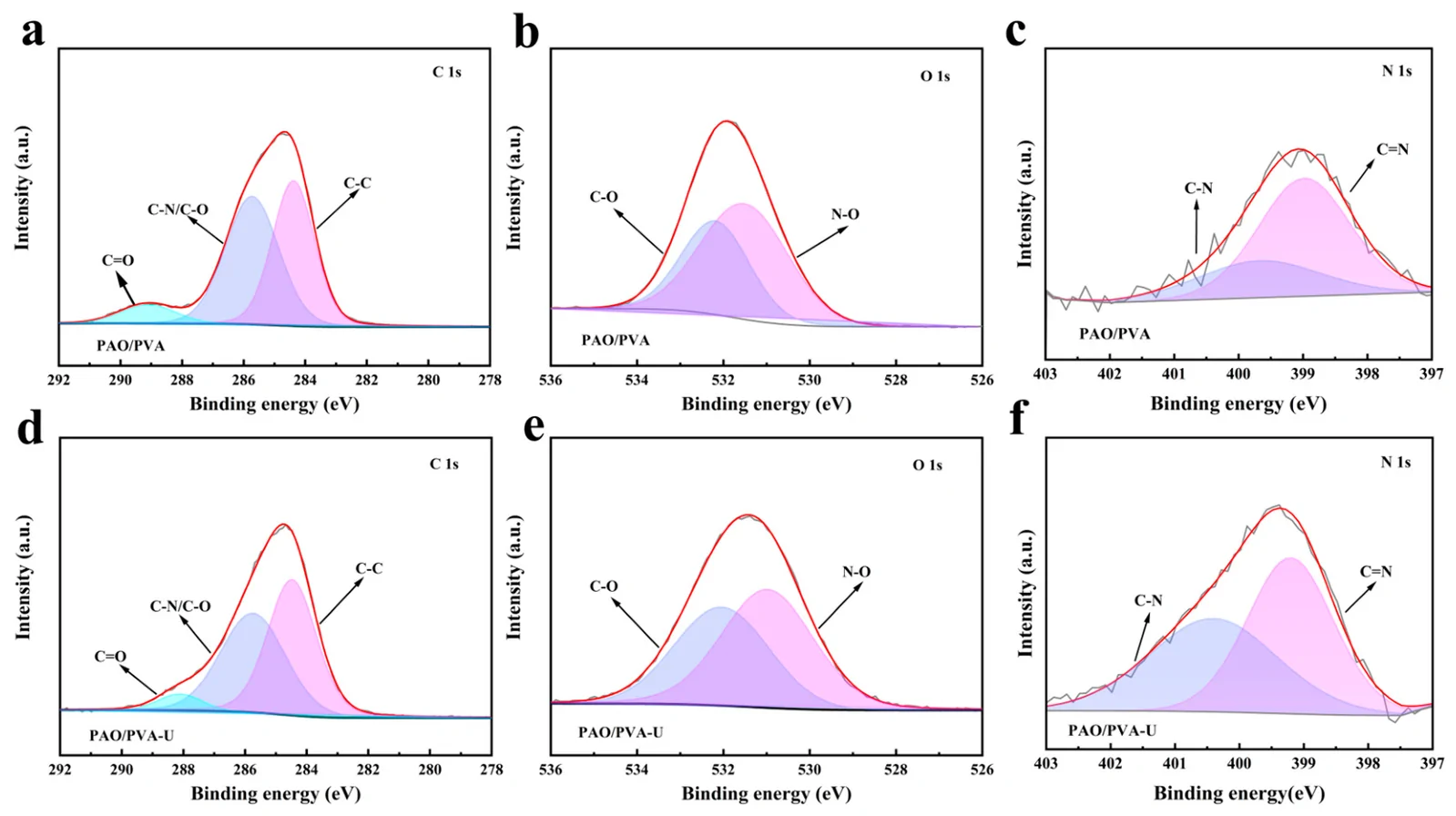
High-resolution (**a**) C 1s, (**b**) O 1s and (**c**) N 1s spectra of PAO/PVA before U(VI) adsorption. (**d**–**f**) The corresponding spectra obtained after U(VI) adsorption.

Figure 8 further compares the high-resolution spectra of C 1s, O 1s and N 1s before and after U(VI) adsorption. The C 1s spectrum of PAO/PVA was deconvoluted into three peaks at 284.4 eV, 285.7 and 288.9 eV (Figure 8a), corresponding to the C-C, C-N/C-O and C=O bonds, respectively. The relative intensity of the C-N/C-O bond decreased dramatically after U(VI) adsorption (Figure 8d), which could be attributed to the coordination interaction between uranyl species and N/O donor atoms. As seen in the high-resolution O 1s spectrum (Figure 8b), the two peaks at 531.5 and 532.3 eV were associated with the oxygen-containing species in PVA and amidoxime groups. The peak positions of N-O and C-O shifted to the lower binding energy after adsorption (Figure 8e), suggesting the participation of oxygen sites in U(VI) binding. As shown in Figure 8c, the binding energies of C=N and C-N in amidoxime-containing polymer were identified at 399.0 and 399.8 eV, respectively. The chemical states of N 1s can be seen to have obvious differences after U(VI) adsorption (Figure 8f), which indicated the involvement of nitrogen sites in uranyl coordination.

The above results reveal that chemical coordination between uranyl species and N/O donor atoms of amidoxime groups is the dominant capture mechanism. A minor contribution from physical adsorption might also exist in this system. PVA was principally responsible for the construction of the recoverable physical network and might also modulate the local hydrogen-bonding environment. Additionally, its hydroxyl groups might provide secondary interactions with uranyl species. Therefore, the excellent adsorption performance could be reasonably attributed to the chemical affinity of amidoxime-rich PAO and the structure/transport features of the PVA-containing porous network.

## 4. Conclusions

In summary, physically cross-linked PAO/PVA microspheres with hierarchical porous architecture were successfully fabricated and further used as high-performance adsorbents for U(VI) capture. The optimized PAO/PVA composites exhibited the highest adsorption capacity at pH = 6 and reached adsorption equilibrium within approximately 300 min. More than 85% of the initial adsorption capacity can also be retained after five adsorption–desorption cycles. In simulated seawater containing competing ions, the PAO/PVA microspheres also demonstrated excellent selectivity for U(VI) capture, with the optimal adsorption capacity of 5.06 mg g^−1^, distribution coefficient of 65.71 L g^−1^ and removal efficiency of 77%. FT-IR and XPS measurements further confirmed the strong coordination interaction between uranyl species and N/O donor atoms derived from amidoxime. Encouraging results from this work may stimulate the development of efficient and recoverable adsorbents for U(VI) capture in complex aqueous media.

## Data Availability

The original contributions presented in this study are included in the article/Supplementary Material. Further inquiries can be directed to the corresponding authors.

## Supplementary Materials

The following supporting information can be downloaded at: https://www.mdpi.com/article/10.3390/ma19183950/s1, Figure S1: The ^13^C-NMR spectra of (a) PAO and (b) PAN in DMSO-D6; Figure S2: TGA curves of the PAO/PVA microspheres; Figure S3: Photographs of the PAO/PVA microspheres (a) before and (b) after U(VI) adsorption; Figure S4: Elution efficiency of uranium-loaded PAO/PVA; Figure S5: Thermal redissolution of the PAO/PVA microspheres in hot water at 95 °C; Figure S6: (a) SEM image of PAO/PVA after U(VI) adsorption. (b) The corresponding EDX spectrum; Table S1: The kinetic parameters for U(VI) adsorption; Table S2: The isotherm parameters for U(VI) adsorption; Table S3: A survey of adsorption performance of various AO-based adsorbents for U(VI) capture; Table S4: The initial concentration, adsorption capacity, distribution coefficient and removal rate of various metal ions. Refs [24,25,26,27,28,29,30,31,32,33] are included in Supplementary Materials.
