# Supplementary material for: Porous PAO/PVA Microspheres for Efficient U(VI) Capture
Source: Materials (Basel). 2026 Sep 17;19(18):3950. doi: 10.3390/ma19183950 (PMC13608057; doi:10.3390/ma19183950)
Supplement: Supplementary file 1 [file materials-19-03950-s001.zip › materials-4530658-supplementary.pdf]

Porous PAO/PVA Microspheres for Efficient U(VI) Capture

Zenghui Wan [1], Xun Sun [1], Shuiyuan Zhang [1], Jun Yang [1,2,*], Long Yu [1], Chao Yuan [1,*], Jingshui Xu [1,3,*] and Suhua Wang [1]

[1] Guangdong Provincial Key Laboratory for Green Agricultural Production and Intelligent Equipment, School of Environmental Science and Engineering, Guangdong University of Petrochemical Technology, Maoming 525000, China
[2] School of Chemical Engineering, Guangdong University of Petrochemical Technology, Maoming 525000, China
[3] Guangdong Provincial Key Laboratory of Petrochemical Equipment Fault Diagnosis, School of Material Science and Engineering, Guangdong University of Petrochemical Technology, Maoming 525000, China
* Correspondence: yangjun@gdupt.edu.cn (J.Y.); yuanchao@gdupt.edu.cn (C.Y.); xujs@gdupt.edu.cn (J.X.)

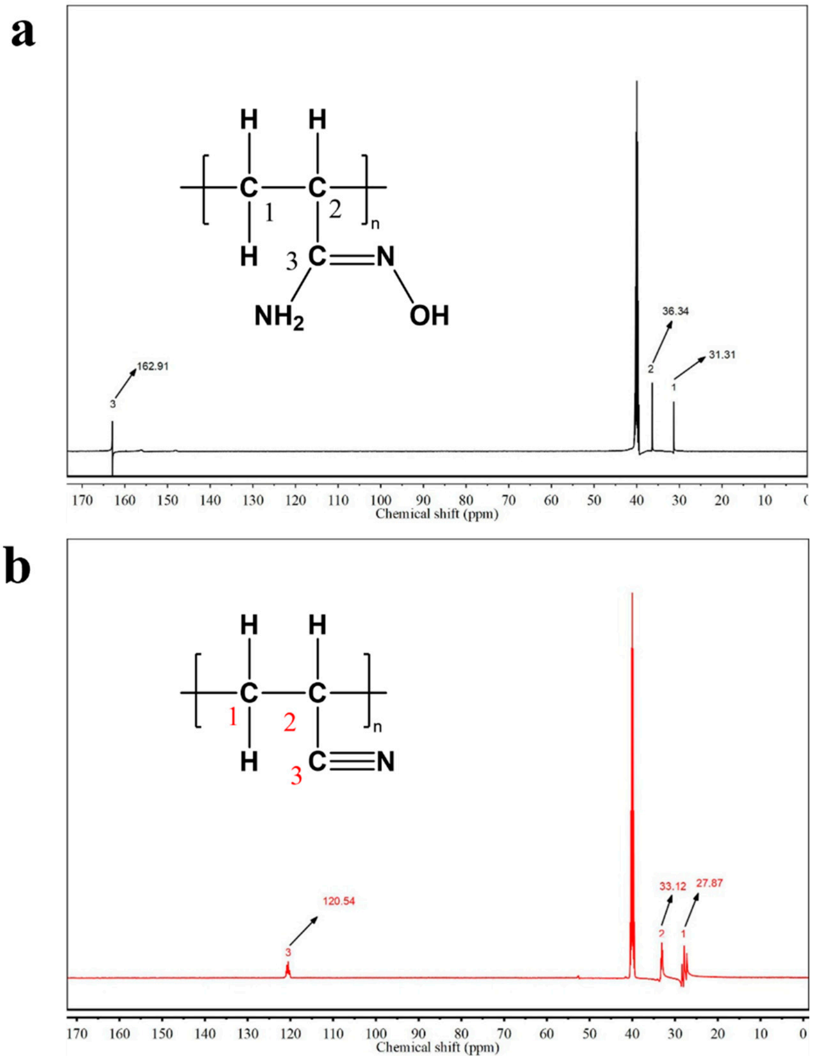

Figure S1. The ^{13}C-NMR spectra of (a) PAO and (b) PAN in DMSO-D6.

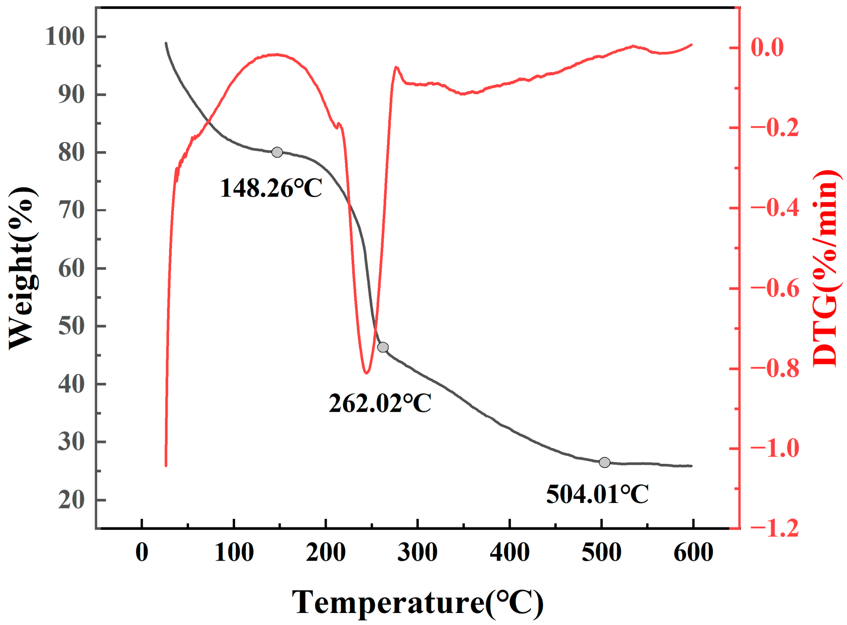

Figure S2. TGA curves of the PAO/PVA microspheres.

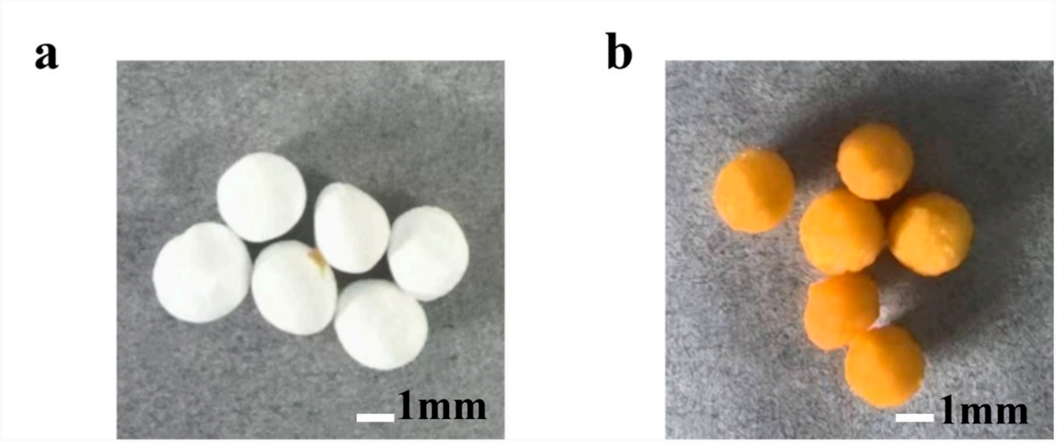

Figure S3. Photographs of the PAO/PVA microspheres (a) before and (b) after U(VI) adsorption.

.

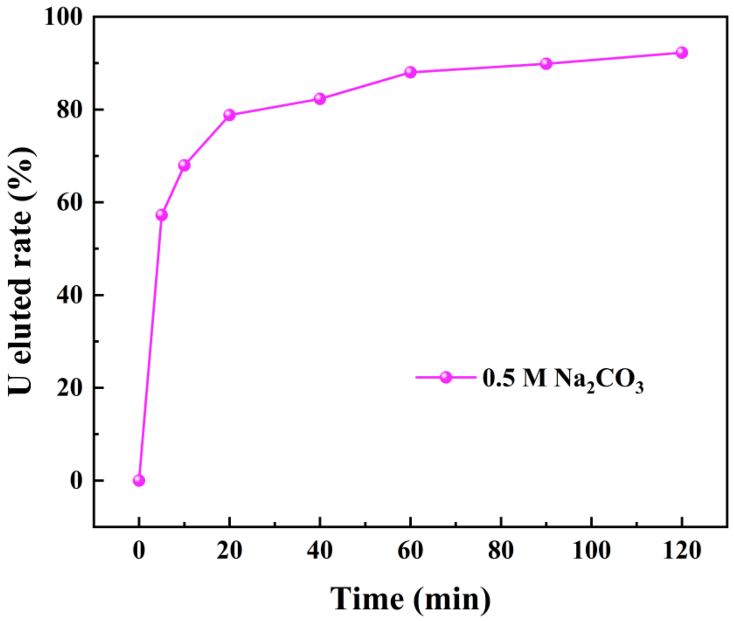

Figure S4. Elution efficiency of uranium-loaded PAO/PVA.

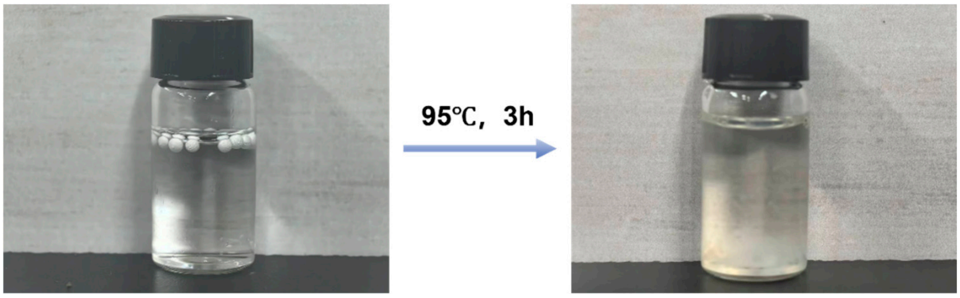

Figure S5. Thermal redissolution of the PAO/PVA microspheres in hot water at 95 °C.

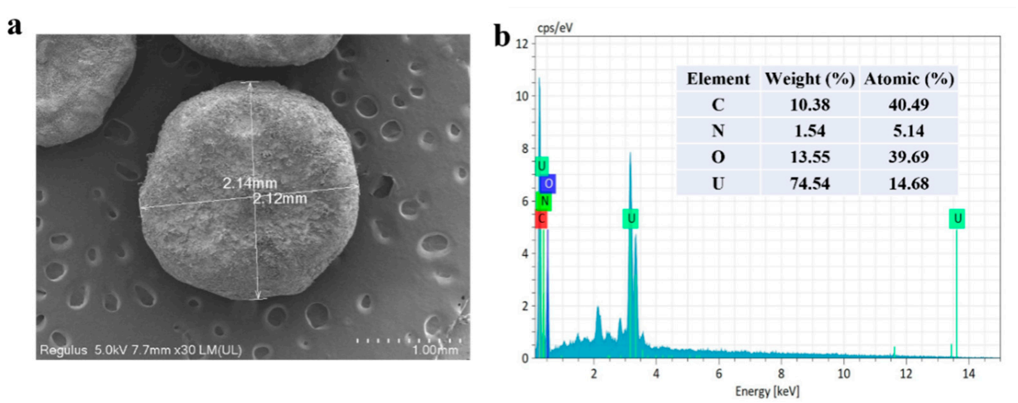

Figure S6. (a) SEM image of PAO/PVA after U(VI) adsorption. (b) The corresponding EDX spectrum.

Table S1. The kinetic parameters for U(VI) adsorption.

Pseudo-first-order	k_1 (min^{-1})	q_e (mg·g^{-1})	R^2
	0.01024	200.97	0.830
Pseudo-second-order	k_2 (g·mg^{-1}·min^{-1})	q_e (mg·g^{-1})	R^2
	0.0000744	217.61	0.916

Table S2. The isotherm parameters for U(VI) adsorption.

Langmuir isotherm	q_m/(mg/g)	K_L/(L/mg)	R^2
	330.16	0.058	0.972
Freundlich isotherm	K_F/(mg/g)/(mg/L)1/n	n	R^2
	46.63	0.423	0.908

Table S3. A survey of adsorption performance of various AO-based adsorbents for U(VI) capture.

Adsorbents	Adsorption conditions	q_e (mg g^{-1})	Ref.
HCPSAN-B-AO	pH = 8, m/V = 1/10 g/L	228.0	[1]
CGPA	pH = 6, m/V=3/10 g/L	263.8	[2]
PCP	pH = 8, m/V = 1/10 g/L	303.8	[3]
HWB-AO	pH=6, m/V=1/2 g/L	256.4	[4]
PAN/PAO	pH = 7.0, m/V = 1/1 g/L	100.2	[5]
PVA-g-AO	pH = 4.0, m/V =2/1 g/L	42.8	[6]
HCP-AO	pH=7.0, m/V =1/5 g/L	234.4	[7]
PP-g-AO	pH=8.0, m/V =1/4 g/L	112.0	[8]
PAN/PVA-AO	pH=6.0, m/V = 1/2.5 g/L	320.6	[9]
ZIF-90-ABOA	pH=8.0, m/V = 1/2.5 g/L	353.4	[10]
HA-PAO NFMs	pH=6.0, m/V = 1/10 g/L	409.0	[11]
PAO/PVA	pH=6.0, m/V = 1/8 g/L	330.2	This work

Table S4. The initial concentration, adsorption capacity, distribution coefficient and removal rate of various metal ions.

Element	natural seawater (ppb)	100×Concentration (ppb)	q_e (mg g^{-1})	K_d (L g^{-1})	E (%)
U	3.3	330	5.06	65.71	76.67
V	2.1	210	2.5	29.41	59.52
Cu	1.4	140	0.6	5.45	21.43
Fe	1.8	180	0.036	0.2	1
Mn	1	100	0.45	5.81	22.5
Ba	14	1400	0.0008	0.00057	0.0029
Zn	4	400	0.742	2.04	9.28
Co	0.05	5	0.0016	0.33	1.6
Pb	0.03	3	0.00123	0.42	2.05
Cr	0.3	30	0.007	0.24	1.17

References

[1] Zhu, L.; Zhang, C.; Qin, F.; Ma, F.; Bi, C.; Zhu, R.; Liu, L.; Bai, J.; Dong, H.; Satoh, T., Amidoxime-modified hypercrosslinked porous poly(styrene-co-acrylonitrile) adsorbent with tunable porous structure for extracting uranium efficiently from seawater. *J. Mol. Liq.* **2022**, *368*, 120741.

[2] Ye, X.; Chi, R.; Wu, Z.; Chen, J.; Lv, Y.; Lin, C.; Liu, Y.; Luo, W., A biomass fiber adsorbent grafted with phosphate/amidoxime for efficient extraction of uranium from seawater by synergistic effect. *J. Environ. Manag.* **2023**, *337*, 117658.

[3] Yu, J.; Zhang, H.; Liu, Q.; Zhu, J.; Yu, J.; Sun, G.; Li, R.; Wang, J., A high-flux antibacterial poly(amidoxime)-polyacrylonitrile blend membrane for highly efficient uranium extraction from seawater. *J. Hazard. Mater.* **2022**, *440*, 129735.

[4] Bai, J.; Li, S.; Yan, H.; Jin, K.; Gao, F.; Zhang, C.; Wang, J., Processable amidoxime functionalized porous hyper-crosslinked polymer with highly efficient regeneration for uranium extraction. *J. Mol. Liq.* **2022**, *362*, 119745.

[5] Xue, Y.; Gui, Y.; Li, Y.; Du, K.; Wang, Y.; Liu, P.; Yan, Y.; Ma, F., An unparalleled wet spinning polyacrylonitrile/polyamidoxime fibers for multi-concentration U(VI) adsorption and elution. *J. Water Process Eng.* **2023**, *54*, 104060.

[6] Chi, F.; Hu, S.; Xiong, J.; Wang, X., Adsorption behavior of uranium on polyvinyl alcohol-g-amidoxime: Physicochemical properties, kinetic and thermodynamic aspects. *Sci. China Chem.* **2013**, *56*, 1495-1503.

[7] Tian, Y.; Wang, Y.; Liu, L.; Dong, H.; Zhu, X.; Ma, F.; Zhang, C., Fabrication of amidoxime functionalized hyper-cross-linked polymer for efficient extraction of uranium(VI) from water. *J. Mol. Liq.* **2023**, *372*, 121171.

[8] Zhang, H.; Zhang, L.; Han, X.; Kuang, L.; Hua, D., Guanidine and amidoxime cofunctionalized polypropylene nonwoven fabric for potential uranium seawater extraction with antifouling property. *Ind. Eng. Chem. Res.* **2018**, *57*, 1662-1670.

[9] Yang, G.; Zhang, Y.; Zhu, Q.; Xia, X.; Pan, N.; Ma, C.; Liu, J.; Liu, Y.; Qin, Y.; Zhang, Q.; Dong, F.; Li, J.; Nie, X., Endeavoring a high amidoxime utilization ratio and adsorption capacity for uranium extraction from seawater: A hydrogen bonding reconstruction strategy. *Adv. Funct. Mater.* **2025**, *35*, 2425281.

[10] Qin, X.; Yang, W.; Yang, W.; Ma, Y.; Li, M.; Chen, C.; Pan, Q., Covalent modification of ZIF-90 for uranium adsorption from seawater, *Micropor. Mesopor. Mat.* **2021**, *323,* 111231.

[11] Shi, S.; Wu, R.; Meng, S.; Xiao, G.; Ma, C.; Yang, G.; Wang, N., High-strength and anti-biofouling nanofiber membranes for enhanced uranium recovery from seawater and wastewater, *J. Hazard. Mater.* **2022**, *436*, 128983.
